# Intraperitoneal Infusion of Mesenchymal Stem/Stromal Cells Prevents Experimental Autoimmune Uveitis in Mice

**DOI:** 10.1155/2014/624640

**Published:** 2014-07-17

**Authors:** Joo Youn Oh, Tae Wan Kim, Hyun Jeong Jeong, Hyun Ju Lee, Jin Suk Ryu, Won Ryang Wee, Jang Won Heo, Mee Kum Kim

**Affiliations:** ^1^Department of Ophthalmology, Seoul National University Hospital, Seoul 110-744, Republic of Korea; ^2^Laboratory of Ocular Regenerative Medicine and Immunology, Seoul Artificial Eye Center, Seoul National University Hospital Biomedical Research Institute, Seoul 110-744, Republic of Korea; ^3^Department of Ophthalmology, Seoul National University Boramae Medical Center, Seoul 156-707, Republic of Korea; ^4^Department of Ophthalmology, Seoul National University College of Medicine, 103 Daehak-ro, Jongno-gu, Seoul 110-799, Republic of Korea

## Abstract

Autoimmune uveitis is one of the leading causes of blindness. We here investigated whether intraperitoneal administration of human mesenchymal stem/stromal cells (hMSCs) might prevent development of experimental autoimmune uveitis (EAU) in mice. Time course study showed that the number of IFN-*γ*- or IL-17-expressing CD4^+^ T cells was increased in draining lymph nodes (DLNs) on the postimmunization day 7 and decreased thereafter. The retinal structure was severely disrupted on day 21. An intraperitoneal injection of hMSCs at the time of immunization protected the retina from damage and suppressed the levels of proinflammatory cytokines in the eye. Analysis of DLNs on day 7 showed that hMSCs decreased the number of Th1 and Th17 cells. The hMSCs did not reduce the levels of IL-1*β*, IL-6, IL-12, and IL-23 which are the cytokines that drive Th1/Th17 differentiation. Also, hMSCs did not induce CD4^+^CD25^+^Foxp3^+^ cells. However, hMSCs increased the level of an immunoregulatory cytokine IL-10 and the population of IL-10-expressing B220^+^CD19^+^ cells. Together, data demonstrate that hMSCs attenuate EAU by suppressing Th1/Th17 cells and induce IL-10-expressing B220^+^CD19^+^ cells. Our results support suggestions that hMSCs may offer a therapy for autoimmune diseases mediated by Th1/Th17 responses.

## 1. Introduction

Autoimmune uveitis of noninfectious origin is a vision-threatening disease that affects 115.3 per 100,000 people and accounts for 2.8–10% of all cases of blindness in the United States [1]. Corticosteroids are the first line of therapy for patients with autoimmune uveitis. However, long-term use of corticosteroids is associated with serious systemic and ocular adverse effects. Also, there are subtypes of autoimmune uveitis that are refractory to steroids [2]. For these reasons, efforts are being made to develop new therapies for autoimmune uveitis by modulating immune responses underlying the pathogenesis of uveitis. The pathogenesis of autoimmune uveitis is traditionally regarded as Th1-mediated [3, 4]. However, Th17 cell is recently identified as a novel subset of T cells that contributes to the development of autoimmune diseases including uveitis [3, 4].

Stromal progenitors of mesodermal cells, referred to as mesenchymal stem cells or multipotent mesenchymal stromal cells (MSCs), have the remarkable capacity to protect tissues from various immune-mediated diseases. Studies demonstrate that MSCs exert immunosuppressive functions by modulating Th1, Th2, or Th17 immune responses, by inhibiting T cell proliferation, or by generating regulatory T (Treg) cells [5–7]. Also, clinical trials are in progress to capitalize on the immunomodulatory effects of MSCs for treatment of patients with autoimmune diseases such as multiple sclerosis, Crohn's disease, type 1 diabetes, systemic lupus erythematous, or systemic sclerosis [8].

In this study, we investigated whether an intraperitoneal (IP) administration of human bone marrow-derived MSCs (hMSCs) might prevent development of experimental autoimmune uveitis (EAU) in mice, a model for human autoimmune uveitis.

## 2. Materials and Methods

### 2.1. Animals

Six-week-old female B6 mice (C57BL/6J) were purchased from Orient Bio Inc. (Seongnam, Korea) and maintained in a specific pathogen-free environment with continuously available water and food. Animals were treated in strict accordance with the ARVO statement for the use of animals in ophthalmic and vision research. The experimental protocols were approved by the Institutional Animal Care and Use Committee of Seoul National University Biomedical Research Institute.

### 2.2. Preparation of hMSCs

Human bone marrow-derived MSCs were obtained from the Center for the Preparation and Distribution of Adult Stem Cells (http://medicine.tamhsc.edu/irm/msc-distribution.html) that supplies standardized preparations of MSCs enriched for early progenitor cells to over 300 laboratories under the auspices of an NIH/NCRR grant (P40 RR 17447-06). Animal experiments were performed with passage two hMSCs from one donor. The cells consistently differentiated into three lineages in culture, were negative for hematopoietic markers (CD34, CD36, CD117, and CD45), and were positive for mesenchymal markers CD29 (95%), CD44 (>93%), CD49c (99%), CD49f (>70%), CD59 (>99%), CD90 (>99%), CD105 (>99%), and CD166 (>99%). The cells were cultured in complete culture medium with 16% FBS until 70% confluence was reached and harvested with 0.25% trypsin/1 mM EDTA at 37°C for 2 min. After washing, the cells were resuspended in balanced salt solution (BSS; BioWhittaker, Walkersville, MD) at a concentration of 10,000 cells/*μ*L for injection* in vivo*.

### 2.3. Induction and Treatment of EAU

EAU was induced in mice by subcutaneous injection into a footpad of 250 *μ*g human interphotoreceptor retinoid binding protein (IRBP) peptide 1–20, GPTHLFQPSLVLDMAKVLLD (20 mg/mL; Peptron, Daejeon, Korea), that was emulsified in complete Freund adjuvant (Sigma, Saint Louis, MO) containing* Mycobacterium tuberculosis* (2.5 mg/mL; BD Difco, Franklin Lakes, NJ). Simultaneously, the mice received 0.7 *μ*g Pertussis toxin (300 *μ*L; Sigma) intraperitoneally. Immediately after immunization, either 1 × 10^6^ hMSCs in 100 *μ*L BSS or BSS (100 *μ*L) alone was injected intraperitoneally.

### 2.4. Histology and Histological Scoring

On days 7, 14, and 21 after immunization, mice were humanely killed, and eyeballs, inguinal and popliteal LNs were collected for further assays. Eyeballs were fixed in 10% formaldehyde and paraffin-embedded. Serial 4 *μ*m thick sections were cut and stained with either hematoxylin/eosin or TUNEL (terminal deoxynucleotidyl transferase dUTP nick end labeling) as the manufacturer's protocol (Chemicon International, Temecula, CA). The morphologic features of the retina were examined, and histological disease scores were assessed by two independent observes (Joo Youn Oh and Tae Wan Kim) in a blinded manner on a scale of 0 to 4 using the criteria previously defined by Caspi [9].

### 2.5. Flow Cytometric Analysis

The proportions of Th1, Th17, *γ* *δ* T, and Treg cells were determined by measuring IFN-*γ*, IL-17, *γ* *δ* TCR, or CD25 and Foxp3-expressing CD4^+^ cells using flow cytometry. In addition, the population of IL-10 expressing cells was determined by staining the cells with CD4, CD25, Foxp3, CD19, B220, CD11b, CD11c, and IL-10. To collect cell suspensions, popliteal or inguinal nodes (LNs) were placed and minced between the frosted ends of two glass slides in RPMI media containing 10% FBS and 1% penicillin-streptomycin. The cells were immunostained with the following fluorescence-conjugated anti-mouse antibodies: CD4, CD25, Foxp3, IFN-*γ*, *γ* *δ* TCR, CD19, B220, CD11b, CD11c, IL-10 (eBioscience, San Diego, CA), and IL-17A (BD Pharmingen, San Diego, CA). For intracellular staining, the cells were stimulated for 4 h with 50 ng/mL phorbol myristate acetate and 1 *μ*g/mL ionomycin in the presence of GolgiPlug (BD Pharmingen). The cells were then assayed for fluorescence using a FACSCanto flow cytometer (BD BioSciences, Mountain View, CA). The gate was set on CD4^+^ or CD19^+^ cell population, and further analysis of surface or intracellular markers was done within this gate. Data were analyzed using Flowjo program (Tree Star, Inc., Ashland, OR).

### 2.6. Real-Time RT-PCR

LNs were lysed in RNA isolation reagent (RNA Bee, Tel-Test Inc., Friendswood, TX) and homogenized using a sonicator (Ultrasonic Processor, Cole Parmer Instruments, Vernon Hills, IL). Total RNA was extracted using RNeasy Mini kit (Qiagen, Valencia, CA) and used to synthesize double-stranded cDNA by reverse transcription (SuperScript III, Invitrogen, Carlsbad, CA). Real-time amplification was performed using TaqMan Universal PCR Master Mix (Applied Biosystems, Carlsbad, CA). An 18 s rRNA probe (TaqMan Gene Expression Assays ID, Hs03003631_g1) was used for normalization of gene expression. For all the PCR probe sets, TaqMan Gene Expression Assay kits were purchased from Applied Biosystems: IL-1*β* (Mm00434228_m1), IL-6 (Mm00446190_m1), IFN-*γ* (Mm01168134_m1) IL-17A (Mm00439618_m1), IL-10 (Mm00439614_m1), TGF-*β* (Mm01178820_m1), IL-12A (Mm00434165_m1), and IL-23 (Mm01160011_g1), and human GAPDH (Hs02758991_g1).

### 2.7. ELISA

For protein extraction, eyeballs were cut into small pieces and lysed in PRO-PREP Protein Extraction Solution (Intron Biotechnology, Seongnam, Korea). The samples were sonicated on ice using an ultrasound sonicator. After centrifugation at 12,000 rpm for 20 min, the supernatant was collected and assayed for IFN-*γ* by ELISA according to the manufacturer's protocol (DuoSet; R & D Systems, Minneapolis, MN).

### 2.8. Statistical Analysis

Values were compared* between* the groups using the one-way ANOVA (SPSS 12.0, Chicago, IL) or Student's *t* test and shown as the mean value ± standard error (SEM). Differences were considered significant at *P* < 0.05.

## 3. Results

### 3.1. hMSCs Reduced Retinal Damage in Mice with EAU

**Figure 1:**
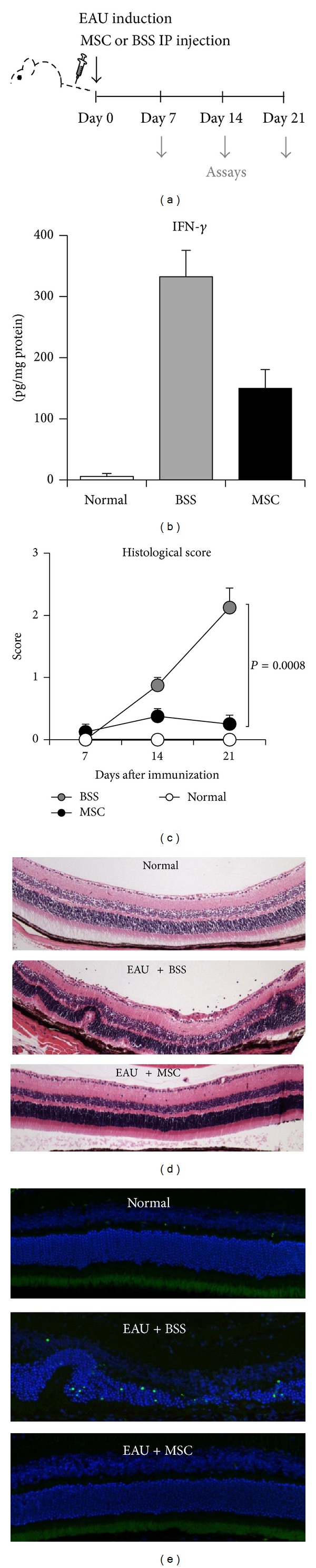
Histological findings of the eye. (a) EAU was induced in mice on day 0, and either hMSCs (1 × 10^6^ cells in 100 *μ*L BSS) or BSS (100 *μ*L) were intraperitoneally (IP) injected immediately after EAU induction. On days 7, 14, and 21, the eyes or DLNs were collected for assays. (b) ELISA showed that IFN-*γ* in the eye was markedly increased in the eyeball on day 14 and significantly reduced by hMSCs. Data are presented in mean + SEM. *n* = 5 in each group. (c) Time course of histological disease scores demonstrated that the retinal pathology gradually developed with a peak at day 21. Histological scores were significantly lower in hMSCs-treated mice at all time-points, suggesting that hMSCs prevented the disease development. Data are presented in mean + SEM. *n* = 5 in each group. (d) Hematoxylin-eosin staining of the eye on day 21 showed severe disruption of the retinal structure including the photoreceptor layer with inflammatory cell infiltration in the vitreous cavity and in the retina of EAU mice. In contrast, the retinal structure was well-reserved, and few inflammatory cells were observed in EAU mice treated with hMSCs. (e) TUENL staining showed a number of dead cells in the disrupted photoreceptor layer of EAU mice. In contrast, no TUENL-positive cells were found in the retina of mice treated with hMSCs.

EAU was induced in mice by subcutaneous injection of IRBP in a footpad on day 0. Simultaneously, either hMSCs (1 × 10^6^ cells/mouse) or BSS was injected intraperitoneally. On days 7, 14, and 21, the mice were humanely killed, and eyeballs and draining LNs (DLNs) were collected for assays (Figure 1(a)). ELISA showed that the level of the proinflammatory cytokine IFN-*γ* was markedly increased in the eyeball on day 14 and significantly reduced by treatment with hMSCs (Figure 1(b)). Histology demonstrated that the retinal structure including the photoreceptor layer was severely disorganized with massive infiltration of inflammatory cells in the vitreous cavity and in the retina of BSS-treated EAU mice on day 21 (histological score 2.13 ± 0.31, Figures 1(c) and 1(d)). In contrast, the retinal architecture was almost completely preserved with few inflammatory cells in hMSCs-treated mice on day 21 (histological score 0.25 ± 0.14, Figures 1(c) and 1(d)). Similarly, TUNEL staining indicated the presence of many dead cells in the photoreceptor layer in BSS-treated EAU mice on day 21, while there were few TUNEL-positive cells in mice treated with hMSCs (Figure 1(e)).

To determine whether hMSCs suppressed the intraocular inflammation by direct contact, we evaluated the presence of hMSCs in the eye by real-time RT-PCR assays for human-specific GAPDH. However, we did not detect any amplification of human GAPDH, indicating that hMSCs were not present in the eye on days 7, 14, or 21 after IP injection.

Therefore, the data clearly indicated that a single IP injection of hMSCs at the time of immunization prevented the development of EAU and protected the retina from inflammation-mediated damage.

### 3.2. hMSCs Decreased Th1/Th17 Cells in DLNs

**Figure 2:**
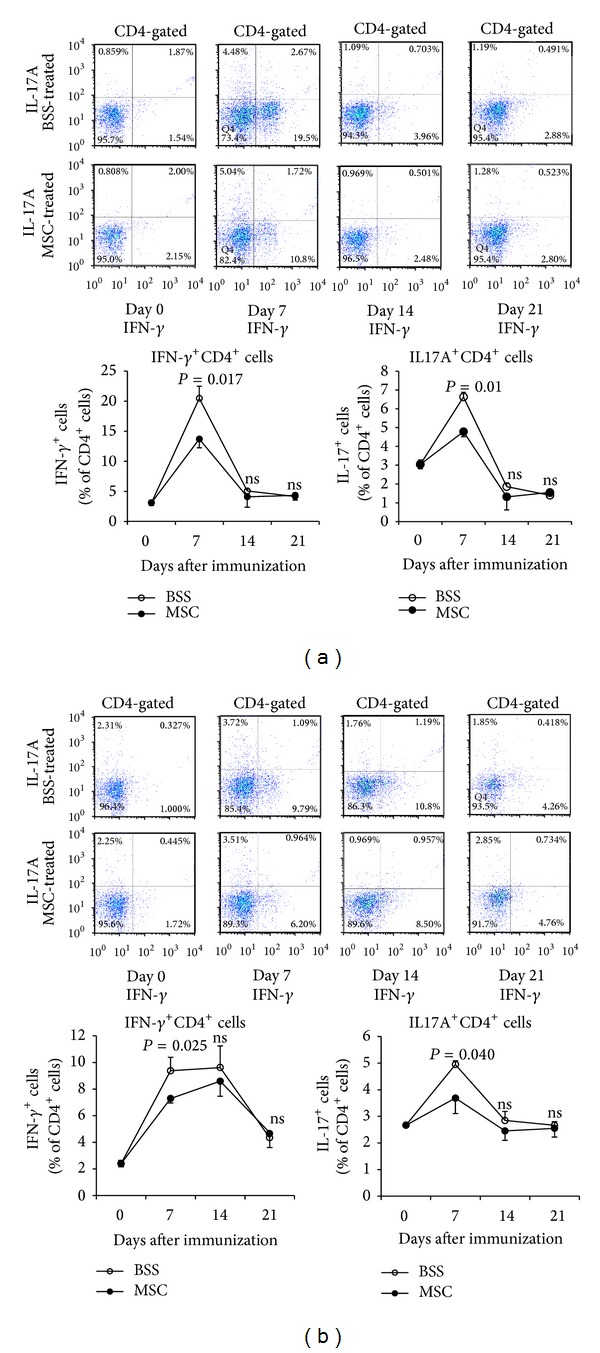
Flow cytometry for Th1 and Th17 cells in LNs. Time course study demonstrated that the percentages of IFN-*γ*-expressing CD4^+^ cells or IL-17A-expressing CD4^+^ cells were significantly increased in popliteal (a) and inguinal LNs (b) on day 7 after EAU induction and decreased thereafter to baseline until day 21. The percentages of both Th1 and Th17 cells were significantly lower in EAU mice treated with hMSCs, compared to BSS-treated mice. Data are presented in mean ± SEM. *n* = 5 in each group.

**Figure 3:**
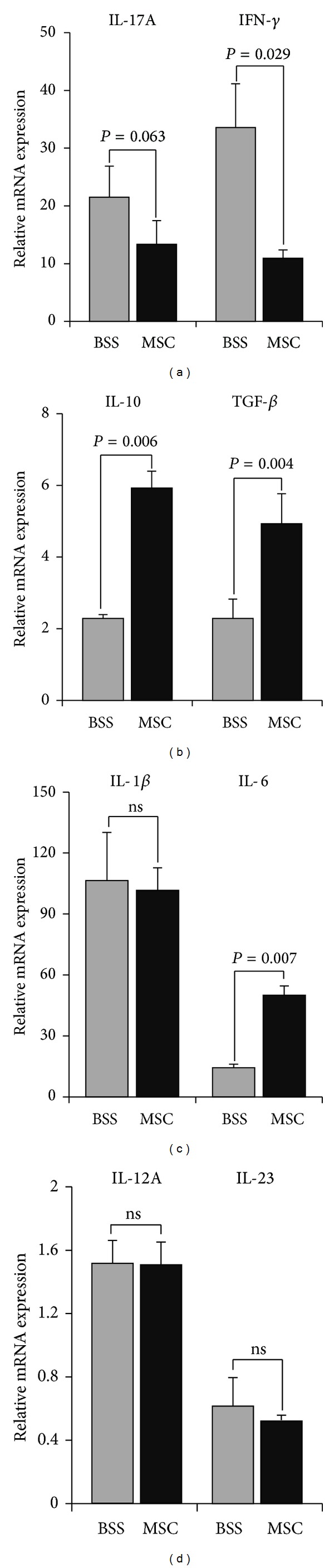
Assay for inflammation- and immune-related cytokines in LNs. Real-time RT-PCR analysis showed that the levels of IL-17A and IFN-*γ* transcripts were increased in popliteal LNs on day 7 after EAU induction and significantly reduced by hMSCs (a). However, the levels of IL-10, TGF-*β*, and IL-6 were significantly increased by hMSCs (b, c). The levels of IL-1*β*, IL-12A, or IL-23 were not different between BSS- and hMSC-treated EAU mice (c, d). Data are presented in mean + SEM. *n* = 5 in each group.

We next investigated whether the tissue-protective effects of hMSCs might be due to the influence of hMSCs on the development of Th1, Th17, or *γ* *δ* T cells that are pathogenic effectors in uveitis [3, 4]. Time course study showed that the percentage of IFN-*γ*-expressing CD4^+^ cells, IL-17A-expressing CD4^+^ cells, and *γ* *δ* TCR-expressing CD4^+^ cells was significantly increased in popliteal and inguinal LNs of EAU mice on day 7 and decreased thereafter to baseline levels (Figure 2, Supplemental Figure 1 (see Supplemental Figure 1 available online at http://dx.doi.org/10.1155/2014/624640)). Treatment with hMSCs significantly reduced the percentage of Th1 and Th17 cells in popliteal and inguinal LNs on day 7 (Figure 2). Consistently, the levels of IL-17A and IFN-*γ* transcripts were significantly reduced by hMSCs, compared to the BSS-treated controls (Figure 3(a)). However, the percentage of *γ* *δ* T cells was not affected by hMSC treatment (Supplemental Figure 1).

In order to determine whether hMSCs inhibited Th1 or Th17 cell generation by suppressing the production of Th1- or Th17-polarizing cytokines, we further measured the levels of IL-1*β*, IL-6, IL-12a, IL-23, and TGF-*β* which are the cytokines that drive the development of Th1 and Th17 cells [10–12]. However, hMSCs did not reduce the levels of IL-1*β*, IL-6, IL-12A, IL-23, and TGF-*β* in DLNs (Figures 3(b)–3(d)). Of note, hMSCs significantly increased the level of IL-10, an immunoregulatory cytokine that suppresses Th1/Th17 immune responses (Figure 3(b)).

### 3.3. hMSCs Induced IL-10-Expressing B220^+^CD19^+^ Cells in DLNs

**Figure 4:**
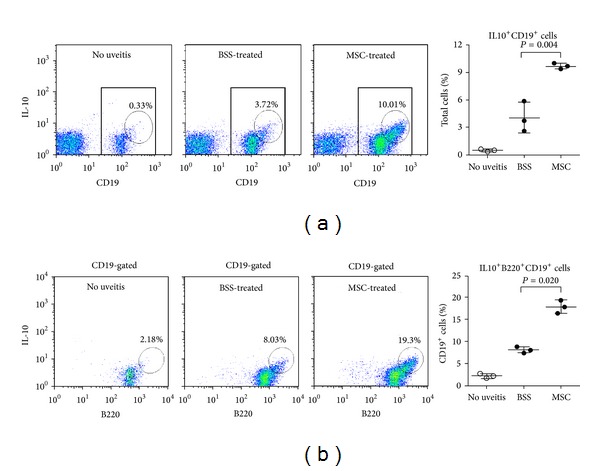
Assay for IL-10-expressing cells in LNs. Flow cytometric analysis of popliteal LNs on day 7 after EAU immunization showed that the percentages of IL-10^+^CD19^+^ (a) and IL-10^+^B220^+^CD19^+^ cells (b) were significantly increased in mice treated with hMSCs, compared to mice without EAU or BSS-treated EAU mice. Data are presented in mean ± SEM. *n* = 5 in each group.

To identify the IL-10-expressing cell population, we examined CD4^+^, CD19^+^, B220^+^, CD11b^+^, or CD11c^+^ cells in DLNs for IL-10 expression. We found that the percentage of IL-10^+^B220^+^CD19^+^ cells was markedly increased in popliteal LNs of hMSCs-treated EAU mice on day 7, compared to BSS-treated EAU mice (Figures 4(a) and 4(b)). However, IL-10 expression was not increased in CD4^+^, CD11b^+^, or CD11c^+^ cells (data not shown). Also, hMSCs did not increase the percentage of CD4^+^CD25^+^Foxp3^+^ Treg cells in popliteal or inguinal LNs at all examined time-points (Supplemental Figures 2(a) and 2(b)). Time course indicated that CD4^+^CD25^+^Foxp3^+^ cells initially increased in popliteal and inguinal LNs on day 7 after EAU induction and gradually decreased to baseline on days 14 and 21. On the contrary, CD4^+^CD25^+^Foxp3^+^ cells initially decreased in the spleen on day 7 and increased thereafter until day 21. The hMSCs significantly suppressed an increase of CD4^+^CD25^+^Foxp3^+^ cells in the spleen on days 14 and 21 (Supplemental Figure 2(c)), reflecting that hMSCs might suppress early inflammation that is required for initial Treg expansion [11].

## 4. Discussion

Time course study revealed that Th1 and Th17 cells increased in DLNs after EAU immunization, and then the levels of proinflammatory cytokines markedly increased in the eyes. Subsequently, the retinal structure was severely and irreversibly disrupted. A single IP administration of hMSCs at the time of immunization significantly decreased Th1 or Th17 cells in DLNs and increased IL10^+^B220^+^CD19^+^ cells on day 7. As a result, ocular inflammation was markedly repressed, and the retina was almost completely protected from damage. Therefore, our data suggest that hMSCs ameliorate autoimmune uveitis by suppressing the Th1/Th17 immune responses.

There are several possibilities that account for the mechanisms of hMSCs in repressing the Th1/Th17 immune responses. One is that hMSCs may directly inhibit differentiation or expansion of Th1/Th17 cells. In support of this hypothesis, previous reports demonstrated that MSCs suppressed T cell proliferation and inhibited differentiation of naïve CD4^+^ T cells into Th1 or Th17 cells [13–18]. However, it is unclear* in vivo* whether enough number of systemically administered hMSCs reaches peripheral LNs or injured tissues to exert direct inhibitory effects on T cells.

Another possibility is that hMSCs may modulate host cells to suppress immune responses. This is likely because a single administration of MSCs that are short-lived has long-term effects in the host as shown in our study. In fact, a number of previous studies showed that MSCs induced Treg cells [18, 19], switched microglia or macrophages toward anti-inflammatory or tissue-repairing phenotypes [20–22], or promoted generation of regulatory or tolerogenic dendritic cells [23–25]. Consistent with these reports, we found that the level of IL-10, an immunoregulatory cytokine that suppresses Th1/Th17 immune responses [26, 27], was markedly upregulated in DLNS of mice treated with hMSCs. Among the examined cell populations (CD4^+^, CD19^+^, CD11b^+^, and CD11c^+^ cells), IL10-expressing B220^+^CD19^+^ cells were dramatically increased. IL-10-producing B cells are known to be critical for suppression of autoimmune diseases, although B cells are both pathogenic and protective in autoimmune diseases [28]. Given that the effects of MSCs on B cells have been rarely studied so far, further characterization of IL10-expressing B220^+^CD19^+^ cells as a potential regulatory B cell subset [29, 30] would provide a mechanistic insight into the immunomodulatory mechanism of hMSCs.

Since the therapeutic efficacy of MSCs was first reported in experimental autoimmune encephalitis [31], there have been efforts to use MSCs for treating a variety of autoimmune diseases such as autoimmune arthritis, autoimmune myocarditis, or Sjögren's syndrome [5, 8, 16, 17, 19]. When it comes to autoimmune uveitis, two studies recently demonstrated that MSCs attenuated EAU in mice and rats. Tasso et al. [32] reported that a single IP injection of syngeneic mouse MSCs at the time of immunization almost completely reduced the incidence and severity of disease in mice with EAU induced by IRBP injection. They found that the percentage of CD4^+^CD25^+^Foxp3^+^ cells was significantly higher in the spleen in MSC-treated EAU mice than in untreated controls. Another study by Zhang et al. [33] reported that an intravenous administration of syngeneic or allogeneic rat MSCs strikingly reduced the severity of EAU induced by IRBP in rats. MSCs were effective when the cells were administered before the onset or at the peak of disease, but not after disease stabilization. They additionally found that the levels of IL-2, IL-17, and IFN-*γ* were lower in supernatants of T lymphocytes isolated from EAU mice treated with MSCs, compared to T lymphocytes from untreated EAU mice. However, either study did not directly evaluate the effects of MSCs on Th1 and Th17 cells in DLNs* in vivo *as in our study.

Another difference of our study is that we used human MSCs for the study. Mouse MSCs undergo spontaneous transformation during expansion in culture and occasionally become tumorigenic in the same manner as mouse fibroblasts [34–36]. Moreover, some of the immunomodulatory effects of MSCs are species-specific. For instance, indolamine 2, 3-dioxygenase (IDO) is involved in the immunosuppressive activity of human MSCs, whereas inducible nitric oxide synthase (iNOS) mediated the immunosuppressive activity of mouse MSCs [37]. Therefore we used hMSCs for this study in order to evaluate clinical efficacy of hMSCs and their mechanism(s) as a potential treatment of autoimmune uveitis in humans. As a result, we found that the number of Th1 and Th17 cells was markedly reduced by hMSCs and IL10^+^B220^+^CD19^+^ cells, not classical CD4^+^CD25^+^Foxp3^+^ Treg cells, were increased by hMSCs. Further studies using a specific depletion of IL-10 production from B cells would be necessary to determine whether IL-10-producing B cells are a main mediator of hMSCs in suppressing EAU.

## 5. Conclusions

In conclusion, our data demonstrate that systemic administration of hMSCs almost completely prevented the development of EAU by suppressing Th1/Th17 immune responses and protected the retina from immune-mediated damage. The results provide a further rationale for the use of hMSCs to treat a variety of autoimmune or immune-mediated diseases involving the eye and other organs that are driven by excessive Th1/Th17 immune responses.

## Supplementary Material

Supplemental figure 1: Flow cytometric analysis for *γ*σ T cells in inguinal and popliteal LNs.
Time course demonstrated that the percentage of *γ*σ TCR-expressing CD4^+^ cells was increased on day 7 in popliteal and inguinal LNs of EAU mice, and decreased thereafter to baseline until day 21. Treatment with hMSCs did not affect the percentage of *γ*σ T cells in either inguinal or popliteal LNs. Data are presented in mean ± SEM. n=5 in each group.
Supplemental figure 2: Assay for CD4^+^CD25^+^Foxp3^+^ regulatory T cells in inguinal and popliteal LNs and in the spleen.
Flow cytometric analysis for CD4^+^CD25^+^Foxp3^+^ cells showed that the percentage of CD4^+^CD25^+^Foxp3^+^ cells was not increased in popliteal or inguinal LNs and in the spleen by hMSCs. However, in the spleen, the percentage of CD4^+^CD25^+^Foxp3^+^ cells was significantly lower in hMSCs-treated group. Data are presented in mean ± SEM. n=5 in each group.
