# Supplementary material for: Intraperitoneal Infusion of Mesenchymal Stem/Stromal Cells Prevents Experimental Autoimmune Uveitis in Mice
Source: Mediators Inflamm. 2014 Jul 17;2014:624640. doi: 10.1155/2014/624640 (PMC4127236; doi:10.1155/2014/624640)

Supplemental figure legends

Supplemental figure 1. Flow cytometric analysis for γδ T cells in inguinal and popliteal LNs.

Time course demonstrated that the percentage of γδ TCR-expressing CD4$^+$ cells was increased on day 7 in popliteal and inguinal LNs of EAU mice, and decreased thereafter to baseline until day 21. Treatment with hMSCs did not affect the percentage of γδ T cells in either inguinal or popliteal LNs. Data are presented in mean ± SEM. n=5 in each group.

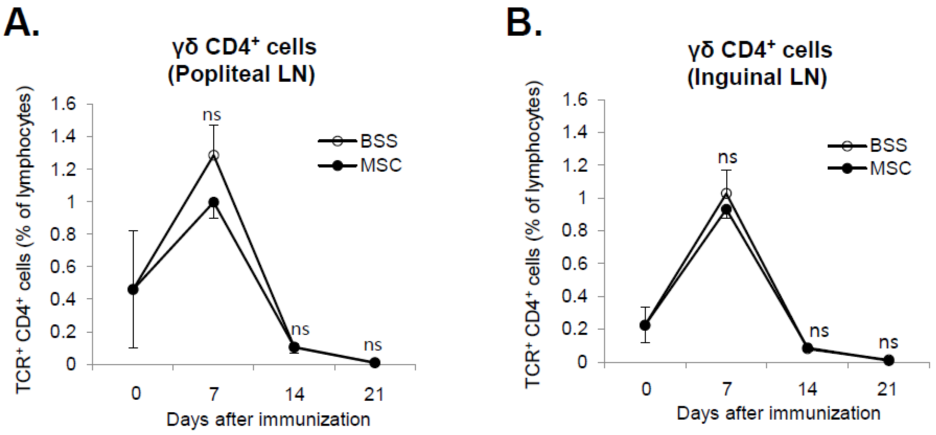

Supplemental figure 2. Assay for $CD4^{+}CD25^{+}Foxp3^{+}$ regulatory T cells in inguinal and popliteal LNs and in the spleen.

Flow cytometric analysis for $CD4^{+}CD25^{+}Foxp3^{+}$ cells showed that the percentage of $CD4^{+}CD25^{+}Foxp3^{+}$ cells was not increased in popliteal or inguinal LNs and in the spleen by hMSCs. However, in the spleen, the percentage of $CD4^{+}CD25^{+}Foxp3^{+}$ cells was significantly lower in hMSCs-treated group. Data are presented in mean ± SEM. n=5 in each group.

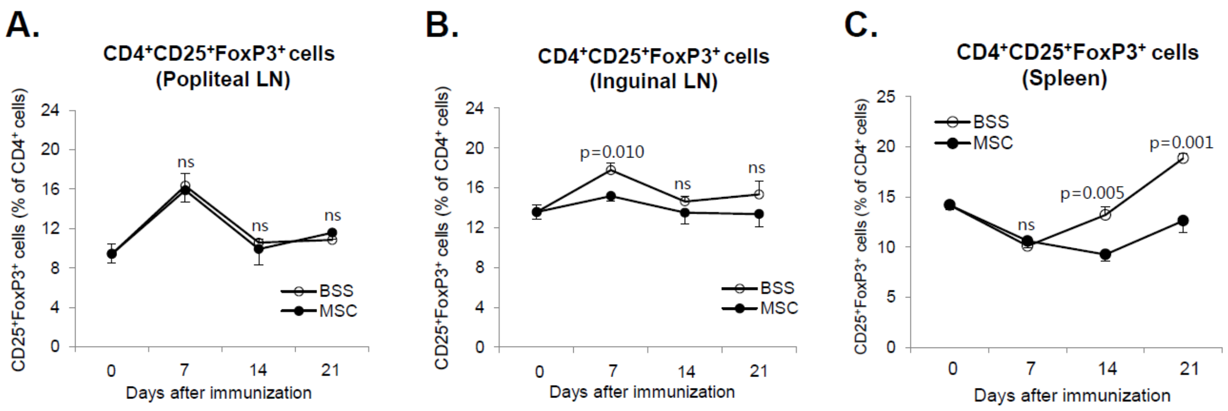

Supplement: Supplementary file 1 — Supplemental figure 1: Flow cytometric analysis for γσ T cells in inguinal and popliteal LNs. Time course demonstrated that the percentage of γσ TCR-expressing CD4+ cells was increased on day 7 in popliteal and inguinal LNs of EAU mice, and decreased thereafter to baseline until day 21. Treatment with hMSCs did not affect the percentage of γσ T cells in either inguinal or popliteal LNs. Data are presented in mean ± SEM. n=5 in each group. Supplemental figure 2: Assay for CD4+CD25+Foxp3+ regulatory T cells in inguinal and popliteal LNs and in the spleen. Flow cytometric analysis for CD4+CD25+Foxp3+ cells showed that the percentage of CD4+CD25+Foxp3+ cells was not increased in popliteal or inguinal LNs and in the spleen by hMSCs. However, in the spleen, the percentage of CD4+CD25+Foxp3+ cells was significantly lower in hMSCs-treated group. Data are presented in mean ± SEM. n=5 in each group. [file 624640.f1.pdf]
